# Acceptability and Feasibility in Using Gamified Mobile Application for Working Memory Training in Older Adults

**DOI:** 10.1002/alz70858_099095

**Published:** 2025-12-24

**Authors:** Frank Ho‐yin Lai, Ben Chi‐bun Yip, Eddie Yip‐kuen Hai, Cath Darling, David Wai‐kwong Man

**Affiliations:** ^1^ The Mental Health Research Centre, The Hong Kong Polytechnic University, Kowloon, Hong Kong; ^2^ Northumbria University, Newcastle, United Kingdom; ^3^ School of Medical and Health Sciences, Tung Wah College, Kowloon, Hong Kong; ^4^ Faculty of Health Sciences and Wellbeing, The Sunderland University, Sunderland, United Kingdom; ^5^ Northumbria Univeristy, Newcastle, Newcastle upon Tyne, United Kingdom; ^6^ School of Medical and Health Sciences, Hong Kong, Hong Kong

## Abstract

**Background:**

Ageing often leads to a decline in working memory, negatively impacting daily functioning and life satisfaction. Gamified mobile applications have emerged as promising instruments for cognitive enhancement, offering the potential to enhance motivation and engagement among the elderly population. This investigation examines the acceptability and practicality of a gamified memory enhancement application involving 20 older people.

**Method:**

Using qualitative methods, the study gathered insights into the preferences and needs of older adults regarding mobile application use. Semi‐structured interviews assessed subjective experiences, perceived usability, and practical utility, while a focus group examined collective perceptions of user satisfaction, barriers to engagement, and reactions to gamified elements. The analyses were assessed through the viewpoint of the Technology Acceptance Model (TAM), underlining perceived benefits and usability, as well as attitudes about and the desire to utilize the application.

**Results:**

Results showed high acceptability, with participants highlighting the application's engaging features and cognitive benefits. However, concerns regarding practicality surfaced, particularly concerning navigation difficulties and the need for clearer instructions. Insights derived from TAM indicated that perceived ease of use and utility had a substantial impact on participants' attitudes.

**Discussion and Conclusion:**

Gamified applications have significant potential for enhancing cognitive function in older adults, but usability improvements are crucial. Consistent with the findings of TAM, ease of use and perceived benefits were pivotal for acceptance, suggesting that improvements focused on user experience could promote wider adoption and sustained engagement.

